# Tunnelling times, Larmor clock, and the elephant in the room

**DOI:** 10.1038/s41598-021-89247-8

**Published:** 2021-05-11

**Authors:** D. Sokolovski, E. Akhmatskaya

**Affiliations:** 1 Departmento de Química-Física, Universidad del País Vasco, UPV/EHU, 48940, Leioa, Spain; 2 Basque Center for Applied Mathematics (BCAM), Alameda de Mazarredo 14, 48009 Bilbao, Bizkaia Spain; 3 IKERBASQUE, Basque Foundation for Science, Plaza Euskadi 5, 48009 Bilbao, Spain

**Keywords:** Quantum mechanics, Attosecond science

## Abstract

A controversy surrounding the “tunnelling time problem” stems from the seeming inability of quantum mechanics to provide, in the usual way, a definition of the duration a particle is supposed to spend in a given region of space. For this reason, the problem is often approached from an “operational” angle. Typically, one tries to mimic, in a quantum case, an experiment which yields the desired result for a classical particle. One such approach is based on the use of a Larmor clock. We show that the difficulty with applying a non-perturbing Larmor clock in order to “time” a classically forbidden transition arises from the quantum Uncertainty Principle. We also demonstrate that for this reason a Larmor time (in fact, any Larmor time) cannot be interpreted as a physical time interval. We provide a theoretical description of the quantities measured by the clock.

## Introduction

The “tunnelling time” problem which has been with us for nearly a century^1^, still has its share of controversy (for a recent review see^2^), and for a good reason. A prerequisite for any constructive discussion is a possibility to define its subject in a meaningful way. For a classical particle, a duration spent in a given region of space is indeed a well established and useful concept. In quantum mechanics, the Uncertainty Principle (UP) in its most general form reads *“one cannot design equipment in any way to determine which of two alternatives is taken, without, at the same time, destroying the pattern of interference”*^3^. In particular, it forbids answering the “which way?” question if two or more pathways leading to the same final outcome interfere. By the same token a duration, even if readily determined for each path, must remain indeterminate for a process where interference between the paths plays a crucial role. This is particularly true in the case of tunnelling.

The early attempts to define the duration a quantum particle spends in the barrier by following the evolution of the transmitted wave packet^4,5^ yielded the so-called Wigner–Smith (WS) time delay, essentially the energy derivative of the phase of the transmission amplitude. One immediate problem with the method is that if the WS result is used to estimate the time spent by the particle in the barrier, this time turns out to be shorter than the barrier width divided by the speed of light. This apparently “superluminal behaviour” does not lead to a conflict with Einstein’s relativity for the simple reason that, in accordance with the Uncertainty Principle, the WS time cannot be interpreted as a physical time interval spent by a tunnelling particle in the barrier^6^. However, as was noted in^2^, the argument of^6^ applies to the “phase time” of^4,5^. Would it still be true if the tunnelling time were defined in a different manner?

An alternative approach was proposed by Baz’^7^, who employed Larmor precession of a magnetic moment (spin) in a magnetic field, small enough not to affect tunnelling seriously^8^. The interest in the Larmor (Baz’) clock was recently renewed after its experimental realisation was reported in^9^ (see also a recent discussion in^10^), and in what follows we will analyse it in some detail. By construction, such a clock probes the response of a scattering amplitude to a small variation of the potential, rather than to a variation of the particle’s energy. Thus, the Larmor time was found to disagree with the Wigner and Smith result, and proposed to be the “correct” estimate of the duration of a scattering process (see the footnote on p. 169 of^11^). Despite Baz’s assertion in^11^, the Larmor clock approach soon encountered its own difficulties. In particular, if applied to tunnelling transmission the method yielded not one but two time parameters, which Büttiker^12^ proposed to combine into a single “interaction time.” In^13^ Sokolovski and Baskin have shown the two Larmor times to be the real and imaginary parts of a “complex time” obtained as an average, in which the usual probabilities were replaced with quantum probability amplitudes. The appearance of a complex quantity, where one would expect a real valued answer, has long caused unease (see for example^14^). It does indeed point to a more fundamental problem, a one which requires further attention.

The purpose of this paper is to demonstrate that the difficulty in deducing the duration spent in the barrier, evident in the analysis of the Wigner–Smith time delay^6^, persists also in the conceptually different Larmor clock approach^7–13^. To do so we will look at a two-component Larmor (Baz’) clock, similar to the one employed in^9^, and appeal to the Uncertainty Principle, a rule of primary importance for any discussion of the tunnelling time problem, yet rarely mentioned in such discussions. It will also be upon us to answer the question “does a Larmor clock measure a physical time interval and, if not, then what does it measure?”

## Results

**Figure 1:**
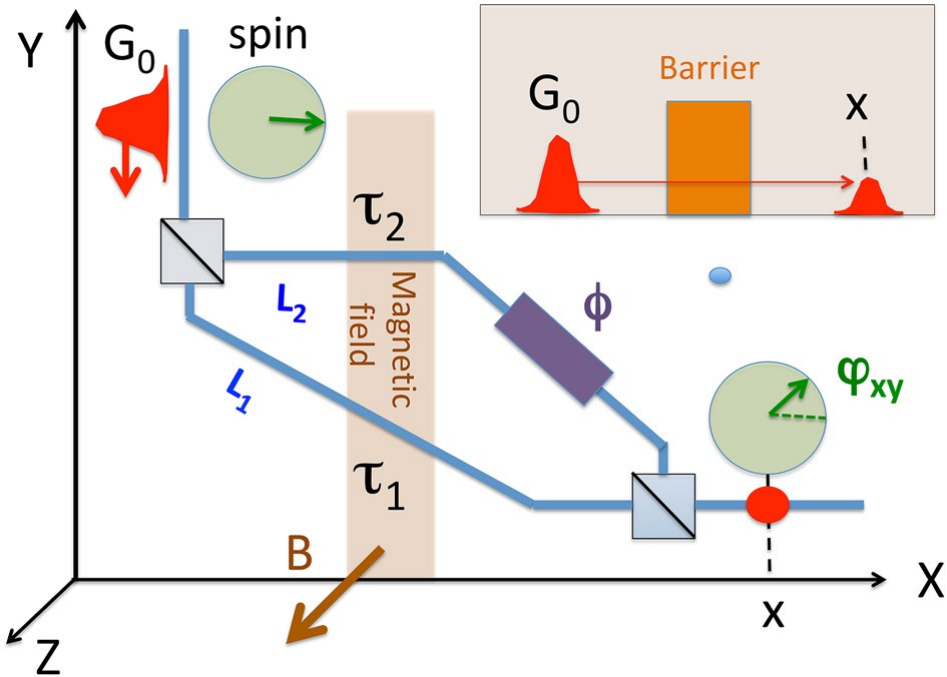
A particle reaches the final position *x* after passing through an interferometer, and a weekly coupled Larmor clock is used to determine the duration it spends in the magnetic field. The case of tunnelling across a potential barrier, shown in the inset, is more complicated, yet conceptually similar.

To lay bare the conceptual difficulty, we start by considering a simple thought experiment, where an electron, with its spin polarised along the *x*-axis, enters an interferometer shown in Fig. 1 in a wave packet state $|G_0{\rangle }$, and is detected after exiting the second beam splitter, as shown in Fig. 1. Travelling via different arms of the interferometer, the electron spends different durations, $\tau _1$ and $\tau _2$, in a region containing constant magnetic field directed along the *z*-axis, *B* (in an experiment using photons and Faraday’s rotation the field would be directed along the arms). An additional element (e.g., an extra potential) in the second arm ensures that an extra phase, $\phi$ is acquired there by both spin components. So how much time did the electron spend in the magnetic field?

The question is more difficult that it may seem. If the wave packet travelling at a velocity *v* is fast, and the field is not too strong, the two spin components acquire, in each arm, phases $\exp (\pm \omega _L\tau _{1,2})$, where $\omega _L$ is the Larmor frequency. Thus, beyond the second beam splitter the wave function is given by (the ${\hat{\sigma }}$s are Pauli matrices)

1 $$\begin{aligned} {\langle }x|\Phi {\rangle }= & {} [G_1(x,t)\exp (-i\omega _L{\hat{\sigma }}_z \tau _1) \nonumber \\&+ G_2(x,t)\exp (-i\omega _L{\hat{\sigma }}_z \tau _2)]|\uparrow _x{\rangle }, \end{aligned}$$

where $G_{1,2}(x,t)$ are the parts of the original wave packets arriving at *x* via the first and the second arm, respectively. One notes that the sum of the rotations in the square brackets does not add up to a single rotation around the *z*-axis, so no duration can be deduced from Eq. (1) directly. Perhaps, making the field small could help? Indeed, sending $\omega _L\rightarrow 0$ and keeping only the linear terms, one finds ${\langle }x|\Phi {\rangle }\approx [G_1(x,t)+G_2(x,t)] (1 -i\omega _L\hat\sigma _z {\overline{\tau }})|\uparrow _x{\rangle }$, which now looks like an overall rotation through a small angle $\omega _L{\overline{\tau }}$. Does this mean that

2 $$\begin{aligned} {\overline{\tau }}\equiv \frac{\tau _1G_1(x,t)+\tau _2G_2(x,t)}{G_1(x,t)+G_2(x,t)}\equiv \tau _1\alpha (\tau _1)+\tau _2\alpha (\tau _2) \end{aligned}$$

is a suitable candidate for the duration spent in the field? Not quite so. The quantities $G_{i}$ are the transition amplitudes^3^ for an electron, initially in $|G_0{\rangle }$, to reach $|x{\rangle }$ via the *i*-th arm of the interferometer, and ${\overline{\tau }}$ is complex valued. This new problem can be dealt with by evaluating the mean angle of precession in the *xy*-plane, $\varphi _{xy}$, guaranteed at least to be real. The result,

3 $$\begin{aligned} \varphi _{xy}\approx {\langle }\Phi |x{\rangle }{\hat{\sigma }}_y{\langle }x|\Phi {\rangle }=\omega _L{\text {Re}}[{\overline{\tau }}], \end{aligned}$$

appears to give preference to the real part of ${\overline{\tau }}$, and may look satisfactory. (Note that measuring the angle of rotation in the *xz*-plane would yield also the value of ${\text {Im}}[{\overline{\tau }}]$, but it is not important to us here.)

However, our real problems are only beginning. A *non-negative* probability distribution, $\rho (z) \ge 0$, has many useful properties. For example, an expectation value ${\langle }z{\rangle }$ marks roughly the centre of the region where $\rho (z)\ne 0$, and the variance gives an estimate of the size of this region. This is no longer true for the distributions which change sign, and the “average” in Eq. (2) is of this latter type. Adjusting the phases and lengths, one can ensure that $G_2(x,t)\approx -G_1(x,t)$, and make the denominator in Eq. (2) small. A similar cancellation will not occur in the numerator, and ${\overline{\tau }}$ can be made as large as one wants. On the other hand, with both arms of about the same length, $L _1\approx L_2\approx L$, the electron spends in motion approximately $\sim L/v$. Now the “duration” in Eq. (3) can easily exceed the total time electron was in motion,

4 $$\begin{aligned} {\text {Re}}[{\overline{\tau }}]>>L/v\equiv T_{total}. \end{aligned}$$

Similarly, $\tau _{1,2}$ and $G_{1,2}$ could be chosen so that ${\overline{\tau }}=0$, making it look like the electron, known to move at a speed *v* in each arm, crosses the field infinitely fast if both arms are considered together. These are serious issues, which should not be ignored. One has to decide whether to allow a quantum particle to spend more time that it has at its disposal, and hail Eq. (4) as a new triumph of quantum theory. The other possibility is to conclude that something is wrong with the very question asked. It is, indeed, frustrating to have two durations, $\tau _1$ and $\tau _2$, and to be unable to combine them into anything meaningful if a particle passes through both arms of the interferometer in Fig. 1.

The frustration is of a familiar kind. In a Young’s double-slit experiment, an electron passes trough one of the two slits, but it is not possible to know which particular slit was chosen. The impossibility of answering the “which way?” question, without destroying interference, is the essence of the Uncertainty Principle, without which quantum mechanics *“would collapse”*^3^. The experiment in Fig. 1 is a kind of a double-slit case, with the only difference that the “which way?” question has been disguised as a “how much time?” query.

It is instructive to see how quantum mechanics implements the Principle in practice. Since the only *a priory* restriction on the in general complex valued *relative amplitudes* $\alpha _{1,2}$ in Eq. (2) is that they should add up to unity, $\alpha _1+\alpha _2=1$, one can find suitable $\alpha$s for *any* choice of a complex ${\overline{\tau }}$,

5 $$\begin{aligned} \alpha _1=\frac{{\overline{\tau }}-\tau _2}{\tau _1-\tau _2}, \quad \alpha _2=-\frac{{\overline{\tau }}-\tau _1}{\tau _1-\tau _2}. \end{aligned}$$

Unable to forbid one to ask the question operationally, quantum theory gives all possible answers, suitable and unsuitable, according to the circumstances. Depending on the parameters of the interferometer, the measured real part of ${\overline{\tau }}$ can be positive, negative, zero, coincide with $\tau _1$ or $\tau _2$, or lie between them. The answer to a question that should not have an answer can be *“anything at all”*.

One can envisage a following dialogue between an experimentalist Alice (${\mathbf{A }}$) and a theoretician Bob (${\mathbf{B }}$).

$${\mathbf{A }}$$:
I have just measured the mean angle $\varphi _{xy}$, and divided it by the Larmor frequency. It follows that $\tau _{Alice}={\text {Re}}[{\overline{\tau }}]= (\tau _1+\tau _2)/2$, a perfectly reasonable result. And I was told this time does not exist.
$${\mathbf{B }}$$:
It does not. Change your settings, and the same procedure will give you ${\text {Re}}[{\overline{\tau }}] <0$. The time parameter you measure is not a meaningful duration.
$${\mathbf{A }}$$:
Let us just forget about the cases where something goes wrong. Surely, in my case it *is* the time an electron spends in the magnetic field.
$${\mathbf{B }}$$:
Just don’t tell that Carol-the-engineer. What she wants, is a time scale for changing the setup slowly enough for the electron “to see” its conditions “frozen” during its journey to the detector. For your $\tau _{Alice}$ to serve as a classical time scale you would also need to show that $\tau _1^n \alpha (\tau _1)+\tau _2^n \alpha (\tau _2)={\overline{\tau }}^n$, $n=1,2,\ldots$. However, this happens only if one of the $\alpha$s vanishes, in which case either $\tau _1$ or $\tau _2$ is the time scale Carol would be happy with.

$${\mathbf{A }}$$:
But this time scale is a very well known and useful concept. How can it not exist?
$${\mathbf{B }}$$:
It is also an essentially classical concept, useful when there is no interference involved. Make one arm of the interferometer much longer than the other, so that the two parts of the wave packet do not overlap at *x*. Then, at a given *t*, you will know which way the electron has travelled, and also the duration, $$\tau _1$$ *or* $$\tau _2$$ it has spent in the magnetic field. But then, of course, it would be a *different* experiment.
$${\mathbf{A }}$$:
And what if I take instead the imaginary part, or the modulus of $${\overline{\tau }}$$, as was suggested, for example by Büttiker^12^?
$${\mathbf{B }}$$:
Or any real valued combination of $${\text {Re}}[{\overline{\tau }}]$$ and $${\text {Im}}[{\overline{\tau }}]$$. You will still encounter “times” which are too long for common sense or too short for Einstein’s relativity, although with $$\tau _{Alice} =|{\overline{\tau }}|$$ you would not need to worry about negative durations.
$${\mathbf{A }}$$:
So what is my “time” good for?
$${\mathbf{B }}$$:
It does describe the response of the electron to a small perturbation of a *particular type*, a small rectangular potential, introduced by the constant magnetic field. A different “time” would arise if the response to a small oscillating potential were to be studied instead^15^.
$${\mathbf{A }}$$:
So, if my time is not a “meaningful duration”, what is it? It looks like one of the “weak values” we heard so much about recently^16^.
$${\mathbf{B }}$$:
It is just what Eq. (3) says, $${\overline{\tau }}$$ is a sum of relative probability amplitudes for reaching the detector via different arms, multiplied by the corresponding durations spent in the field, thus, also an amplitude. And so is every other “weak value”^17^. Your time is just the real part of a particular probability amplitude.
$${\mathbf{A }}$$:
But I have just measured it.
$${\mathbf{B }}$$:
Not quite, you just measured the spin, and then tried to learn something about electron’s translational degree of freedom. In doing so, you relied on the first-order perturbation theory. Response of a system to a small perturbation is commonly described in terms of real valued combinations of the system’s probability amplitudes.
$${\mathbf{A }}$$:
And what is then an amplitude?
$${\mathbf{B }}$$:
According to Feynman^3^, it is a basic concept in our description of quantum behaviour.
$${\mathbf{A }}$$:
This does not tell me very much. Can you be more specific?
$${\mathbf{B 
}}$$:
I am afraid not. Nor, I suspect, can anybody else, unless a radically new insight into physics of the double-slit experiment is gained in future. In Feynman’s words, at the moment *“no one will give you any deeper description of the situation”*^3^.

The case of Ref.^9^ is similar to the one just discussed, if not more involved (see “Methods”). In Fig. 1, there are only two routes by which an electron, starting in a state $|G_0{\rangle }$, can reach the final position *x*, and the corresponding amplitude has two components,

6 $$\begin{aligned} A(x\leftarrow G_0)= & {} G_1(x,t)+G_2(x,t) \nonumber \\\equiv & {} A(x\leftarrow G_0|\tau _1)+A(x\leftarrow G_0|\tau _2). \end{aligned}$$

For a quantum particle crossing a potential barrier, there are many possible $\tau$s, and many components to the transition amplitude^18^,

7 $$\begin{aligned} A(x\leftarrow G_0)=\int _0^{T_{total}} A(x\leftarrow G_0|\tau ) d\tau . \end{aligned}$$

The mean angle of spin’s rotation in a small magnetic field, confined to the barrier, is given by an analogue of (3)

8 $$\begin{aligned} \varphi _{xy}\approx & {} \omega _L{\text {Re}}\left[ \frac{\int _0^{T_{total}} \tau A(x\leftarrow G_0|\tau ) d\tau }{\int _0^{T_{total}} A(x\leftarrow G_0|\tau ) d\tau } \right] \quad \nonumber \\= & {} \omega _L{\text {Re}}\left[ {\int _0^{T_{total}} \tau \alpha (\tau ) d\tau }\right] \equiv \omega _L{\text {Re}}\left[ \overline{\tau }\right] . \end{aligned}$$

In the classical limit, highly oscillatory $A(x\leftarrow G_0|\tau )$ develops a stationary region around the classical duration $\tau _{class}$, where it varies more slowly. This is the only region contributing to the integral in (8), and one recovers the classical result, ${{\overline{\tau }}}=\tau _{class}$. But this well defined duration disappears already if $A(x\leftarrow G_0|\tau )$ has two, rather than just one, stationary regions, and we are back to the situation similar to the one shown in Fig. 1.

Quantum *tunnelling* is a destructive interference phenomenon, where $A(x\leftarrow G_0|\tau )$ in Eq. (6) has no stationary regions, and rapidly oscillates throughout the allowed range $0 \le \tau \le T_{total}$. The tunnelling amplitude (6) is extremely small for a tall or a wide barrier (see the inset in Fig. 1). This happens not because $A(x\leftarrow G_0|\tau )$ is itself small, but because its oscillations cancel each other almost exactly. The delicate balance is easily perturbed, and an attempt to destroy interference between different durations would also destroy the tunnelling transition one wanted to study.

## Discussion

Finally, if Alice were to repeat also the experiment of Ref.^9^, this is what Bob would say about her result. “A fundamental problem, arising each time a Larmor clock is applied to tunnelling, but often overlooked - the proverbial elephant in the room - has to do with the quantum Uncertainty Principle. According to the Principle, one can have tunnelling, and not know the time spent in the barrier, or know this duration, but have tunnelling destroyed. One faces precisely the same choice in the double slit experiment, where he/she must decide between knowing the slit chosen by the particle, or having the interference pattern on the screen, but not both at the same time. You have tried to keep tunnelling intact (your clock perturbs it only slightly), and learn something about the duration spent in the barrier. You might expect the UP to make your result *always* look flawed in one way or another, but this is not how the UP works. If you consider all possible experiments of this type, some of them will give seemingly reasonable outcomes, whereas other ’times’ would be negative, too short, too long, etc. This is necessary, and is possible because such ’times’ can be expressed as the combinations of probability amplitudes which, unlike probabilities, have few restrictions on their signs and magnitudes. Though your result of 0.61 *ms* does look plausible you cannot recommend using it the way you would use a classical time scale just because of this. After all, in a double-slit experiment one cannot cherry pick the points on the screen, where the ’which way?’ question can be answered meaningfully, since the Uncertainty Principle applies everywhere in equal measure. You cannot say that you resolved the controversy regarding how long a tunnelling particle spends in the barrier region, or proved that this duration is non-zero. The controversy, if you wish to call it that, goes to the very heart of the quantum theory, and must be accepted, rather than resolved.”

## Methods

### Probability amplitude to spend a given duration $\tau$ in the barrier

Consider a particle with a mean momentum $p_0$, prepared in a wave packet state $G_0$ ($\hbar =1$),

9 $$\begin{aligned} G_0(x) = \int {a}(p-p_0)\exp (ipx)dp= \exp (ip_0x){W(x)}, \end{aligned}$$

where ${a}(p-p_0)$ describes the distribution of the particle’s momenta, and *W*(*x*) is the wave packet’s envelope. At $t=0$ the wave packet lies to the left of a potential barrier *V*(*x*) of a width *d*, as shown in the inset in Fig. 1. All momenta *p* in (9) are such that in order to cross the barrier the particle has to tunnel. The probability amplitude to detect the particle at *x* close to the maximum of the transmitted wave packet, after it has been in motion for $T_{total}$ seconds, can be represented as a sum over Feynman paths,

10 $$\begin{aligned} A(x\leftarrow G_0) = \int dx' \sum _{paths} \exp (iS[x(t)]G_0(x'), \end{aligned}$$

where a path *x*(*t*) starts in $x'$ at $t=0$, and ends in *x* at $t=T_{total}$. The action functional is given by the usual $S[x(t)]=\int _0^{T_{total}}[{\mu {\dot{x}}^2}/{2}-V(x)]dt$, with $\mu$ denoting the particle’s mass. Each path spends a certain amount of time in the barrier region $0\le x\le d$. Thus duration can be computed with the help of a “stop-watch” (SW) expression,

11 $$\begin{aligned} {\tau _{SW}}[x(t)]=\int _0^{T_{total}} \theta _{[0,d]}(x(t)) dt, \end{aligned}$$

where $\theta _{[0,d]}=1$ for $0\le x\le d$ and 0 otherwise, so that only the time intervals spent in the barrier are added to the total. It is readily seen that ${\tau _{SW}}[x(t)]$ cannot be negative, nor can exceed the time the particle was in motion, hence

12 $$\begin{aligned} 0\le {\tau _{SW}}[x(t)]\le T_{total}. \end{aligned}$$

A simple cosmetic operation turns the path sum (10) into the sum over durations spent in the barrier. Restricting the summation to the paths which spend there precisely $\tau$ seconds, yields

13 $$\begin{aligned} A(x\leftarrow G_0|\tau ) \equiv \int dx' \sum _{paths} \delta ({\tau _{SW}}[x(t)]-\tau ) \exp (iS[x(t)]G_0(x'), \end{aligned}$$

where $\delta (z)$ is the Dirac delta, and we have

14 $$\begin{aligned} A(x\leftarrow G_0) \equiv \int _0^{T_{total}}A(x\leftarrow G_0|\tau )d\tau . \end{aligned}$$

This is bad news for one’s effort to determine the time *actually* spent in the potential - all such durations interfere. We are back to the Young’s interference experiment, except that instead of two paths, each going through one of the slits, we have a continuum of routes, each labelled by the value of the ${\tau _{SW}}[x(t)]$. According to the Uncertainty Principle^3^ the “which way?” ( “which $\tau$?”) question has no answer. The only exception is the classical limit. Typically, $A(x\leftarrow G_0|\tau )$ is highly oscillatory, but in a classically allowed case, e.g., with the barrier removed, the oscillations are slowed down near the classical value $\tau _{cl}=\mu d/p$. If $A(x\leftarrow G_0|\tau )$ has a unique *stationary phase point* of this kind, $\tau _{cl}$ will appear as the only time parameter, whenever one evaluates integrals involving $A(x\leftarrow G_0|\tau )$, and classical mechanics will apply as a result.

The problem with tunnelling is that no such preferred time emerges for a classically forbidden transition, and all $\tau$s must be treated equally (a similar situation is shown in Fig. 3 of^6^, although for a different quantity). To make things worse, in tunnelling the amplitude $A(x\leftarrow G_0)$ is very small ($\sim \exp [-(2\mu V-p_0^2)^{1/2}d]$ for a rectangular barrier), while $A(x\leftarrow G_0|\tau )$ is not. Thus, the exponentially small tunnelling amplitude results from a highly accurate cancellation between (not small) oscillations of $A(x\leftarrow G_0|\tau )$. For this reason, any attempt to modify or neglect any part of the integrand in Eq. (13) would considerably change the result, and destroy the tunnelling.

### An uncertainty relation for the duration $\tau$

Although the Uncertainty Principle hampers one’s attempts to ascribe a unique barrier duration to a tunnelling transition^19^, there is still one more thing we can do. Writing the $\delta$-function in (13) as

15 $$\begin{aligned} \delta ({\tau _{SW}}[x(t)]-\tau )= (2\pi )^{-1}\int d\lambda \exp \{i\lambda (\tau -{\tau _{SW}}[x(t)])\}, \end{aligned}$$

and inserting it into (13), we note that the new action

16 $$\begin{aligned} S_\lambda [x(t)] \equiv S[x(t)] -\lambda \int _0^{T_{total}}dt {\theta _{[0,d]}(x(t))} \end{aligned}$$

corresponds to adding to the barrier *V*(*x*) a rectangular potential $\lambda \theta _{[0,d]}(x(t))$, a well or a barrier, depending on the sign of $\lambda$. Equation (13) can now be written in an equivalent form,

17 $$\begin{aligned} A(x\leftarrow G_0|\tau )=(2\pi )^{-1}\int _{-\infty }^{\infty } d\lambda \exp (i\lambda \tau ) {{\tilde{A}}}(x\leftarrow G_0|\lambda ), \end{aligned}$$

where ${{\tilde{A}}}(x\leftarrow G_0|\lambda )$ is the amplitude to reach, at $t=T_{total}$, the final location *x* from the initial state $G_0$, while moving in a combined potential $V(x)+\lambda \theta _{[0,d]}(x)$. In other words, to evaluate the amplitude $A(x\leftarrow G_0|\tau )$ one needs to know the amplitudes of transmission for all composite potentials. And *vice versa*, to know the amplitude for a given potential one needs to know the amplitudes for all durations spent therein.

Note next that even the calculation of the full amplitude distribution of the durations spent in a region [0, *d*] for a free particle, $V(x)=0$, is already a non-trivial task. It involves evaluation of the transmission amplitudes for all rectangular wells and barriers, and integration in Eq. (17). However, once $A_0(x\leftarrow G_0|\tau )$ is obtained, the distribution for a rectangular potential $V(x)=V\theta _{[0,d]}(x)$ comes for free,

18 $$\begin{aligned} A(x\leftarrow G_0|\tau )=\exp (-iV\tau )A_0(x\leftarrow G_0|\tau ). \end{aligned}$$

As we mentioned above, in the semiclassical limit, the free amplitude distribution $A_0(x\leftarrow G_0|\tau )$ develops a stationary region around $\tau =\mu d/p_0$. When the barrier is raised, the factor $\exp (-iV\tau )$ destroys the stationary region, $A(x\leftarrow G_0|\tau )$ rapidly oscillates everywhere, and $A(x\leftarrow G_0)$ becomes small for a tunnelling particle.

Equation (17) is a kind of uncertainty relation between the duration $\tau$ and the potential in the region of interest. It implies that a device employed to measure the $\tau$ must introduce some uncertainty into the potential, the greater the uncertainty, the more accurate the measurement. Which brings us to the Larmor clock.

### The Larmor clock

The clock consists of a magnetic moment, proportional to an angular momentum (spin) of a size *j*, coupled to a magnetic field along the *z*-axis via $\hat{H}_{int}=\omega _L{\hat{j}}_z$, where $\omega _L$ is the Larmor frequency. By the time *t*, an initial state

19 $$\begin{aligned} |\gamma {\rangle }=\sum _{m=-j}^j\gamma _m|m{\rangle }, \quad {\hat{j}}_z|m{\rangle }=m|m{\rangle }\end{aligned}$$

becomes rotated by an angle $\omega _Lt$ around the *z*-axis,

20 $$\begin{aligned} |\gamma (t){\rangle }=\exp (-i\omega _Lt{\hat{j}}_z)|\gamma (0){\rangle }= \sum _{m=-j}^j\gamma _m\exp (-i m\omega _Lt)|m{\rangle }. \end{aligned}$$

Let us suppose the spin travels with a classical particle moving along a trajectory *x*(*t*), and the field exists only in the region $0\le x \le d$. Then the spin, precessing only when the particle is in the field, $0\le x(t) \le d$, ends up rotated by $\omega _L{\tau _{SW}}[x(t)]$ by $t=T_{total}$. Quantally, for a particle in the inset of Fig. 1, the final (unnormalised) spin’s state can be found simply by adding up its states, rotated by $\omega _L\tau$, each multiplied by the probability amplitude of spending in the field a net duration $\tau$. The result is

21 $$\begin{aligned} |\gamma (T_{total}){\rangle }=\int _0^{T_{total}}d\tau A(x\leftarrow G_0|\tau )\exp (-i\omega _L\tau {\hat{j}}_z)|\gamma (0){\rangle }. \end{aligned}$$

In general, the r.h.s. of (21) cannot be rewritten as a single rotation around the *z*-axis by an angle $\omega _L\tau '$, $|\gamma (T_{total}){\rangle }\ne \exp (-i\omega _L\tau ' {\hat{j}}_z)|\gamma (0){\rangle }$, and no unique time $\tau '$ can be associated with a quantum transition in this way.

With the help of Eq. (17), one obtains an equivalent form of Eq. (21),

22 $$\begin{aligned} |\gamma (T_{total}){\rangle }= \sum _{m=-j}^j{{\tilde{A}}}(x\leftarrow G_0|m\omega _L)\gamma _m|m{\rangle }. \end{aligned}$$

This shows that each spin component traverses the barrier as if the potential there were $V(x)+m\omega _L$, so the potential, experienced by the particle as a whole, remains uncertain within the range from $-j\omega _L$ to $j\omega _L$. As was already noted, a viable clock has to introduce this uncertainty, and we may ask what can be learnt about the duration spent in the barrier by applying the Larmor clock.

An experiment could consist in detecting, at $t=T_{total}$, the particle in *x* and its spin in a state $|\beta {\rangle }=\sum _{m=-j}^j\beta _m|m{\rangle }$. From (21) the corresponding probability is

23 $$\begin{aligned} P(\beta , x \leftarrow \gamma ,G_0) = |\int _0^{T_{total}} d\tau \Gamma (\tau |\omega _L,\beta ,\gamma )A(x\leftarrow G_0|\tau )|^2, \end{aligned}$$

where

24 $$\begin{aligned} \Gamma (\tau |\omega _L,\beta ,\gamma )\equiv {\langle }\beta |\exp (-i\omega _L\tau {\hat{j}}_z)|\gamma {\rangle }=\sum _{m=-j}^j\beta ^*_m \gamma _m\exp (-i m\omega _L\tau). \end{aligned}$$

Thus, by measuring the probability (23), one can determine the absolute value of the integral in Eq. (23), which involves the amplitude distribution of the durations spent by the particle inside the barrier in the absence of the clock. Note that little is left of the original tunnelling transition, where the transmission amplitude $A(x\leftarrow G_0)$ is typically small. As already mentioned  in the first subsection of the Methods, the presence of an additional factor such as $\Gamma (\tau |\omega _L,\beta ,\gamma )$ is likely to alter destructive interference which defines tunnelling. As a result, $\int _0^{T_{total}} d\tau \Gamma (\tau |\omega _L,\beta ,\gamma )A(x\leftarrow G_0|\tau )$ could differ from the original tunnelling amplitude in Eq. (14) by orders of magnitude.

### A non-perturbing (weak) Larmor clock

One can try to return to tunnelling by sending $\omega _L\rightarrow 0$, and learn something about the tunnelling time from the particle’s response to the clock. (This already bodes ill for one’s task, since the uncertainty introduced in the potential will also tend to zero, which,   according to the second subsection of the Methods, should lead to a large uncertainty in $\tau$). Nevertheless, we obtain

25 $$\begin{aligned} \Gamma (\tau |\omega _L,\beta ,\gamma ) \approx {\langle }\beta |\gamma {\rangle }- i\omega _L\tau {\langle }\beta |{\hat{j}}_z|\gamma {\rangle }, \end{aligned}$$

so that the relative change in the probability (23) with and without the magnetic field is

26 $$\begin{aligned} \frac{P(\beta , x \leftarrow \gamma ,G_0)_{\omega _L}-P(\beta , x \leftarrow \gamma ,G_0)_{{\omega _L}=0}}{P(\beta , x \leftarrow \gamma ,G_0)_{{\omega _L}=0}} \approx 2 {\text {Re}}[Z(\beta , \gamma )]{\text {Im}}[{{\overline{\tau }}}]+ {\text {Im}}[Z(\beta , \gamma )]{\text {Re}}[{{\overline{\tau }}}], \end{aligned}$$

where $$Z(\beta , \gamma )\equiv {{\langle }\beta |{\hat{j}}_z|\gamma {\rangle }/{\langle }\beta |\gamma {\rangle }}$$ and

27 $$\begin{aligned} {{\overline{\tau }}} \equiv \frac{\int _0^{T_{total}} \tau A(x\leftarrow G_0|\tau ) d\tau }{\int _0^{T_{total}} A(x\leftarrow G_0|\tau ) d\tau } =\frac{\int _0^{T_{total}} \tau A(x\leftarrow G_0|\tau ) d\tau }{A(x\leftarrow G_0)} \equiv \int _0^{T_{total}} \tau \alpha (\tau ) d\tau \end{aligned}$$

is the complex time of Sokolovski and Baskin^13^. The quantity in the l.h.s. of Eq. (27) can be measured, and by choosing a different $|\beta {\rangle }$ one can, in principle, determine the values of ${\text {Re}}[{{\overline{\tau }}}]$, ${\text {Im}}[{{\overline{\tau }}}]$, or indeed of their various combinations. Moreover, for ${\langle }\beta |\gamma {\rangle }=0$, one has

28 $$\begin{aligned} P(\beta , x \leftarrow \gamma ,G_0)_{\omega _L}\sim \omega _L^2|{{\overline{\tau }}}|^2, \end{aligned}$$

so the modulus of ${{\overline{\tau }}}$ can also be determined directly.

Now there are many real valued time parameters related to the complex time (27), yet none of them is a suitable candidate for a physical time interval representing the net duration spent in the barrier. The easiest way to demonstrate it is to note that for an improbable transition, $A(x\leftarrow G_0|\tau ) \rightarrow 0$, the denominator of (27) can be very small. At the same time, the numerator does not have to be small, since multiplication of $A(x\leftarrow G_0|\tau )$ by $\tau$ can destroy the cancellation, characteristic of tunnelling. Thus, $|{{\overline{\tau }}}|$ may, in principle, exceed the total duration of motion, $|{{\overline{\tau }}}|>> T_{total}$. This makes little sense, especially if one recalls that each and every Feynman path in Eq. (10) spends in the barrier no more than $T_{total}$.

### The Baz’ clock

Finally we briefly discuss a particular type of a weak Larmor clock, employing a spin-1/2 in a weak magnetic filed. It was introduced by Baz’ more than 50 years ago^7^, and recently implemented by Ramos et al. in^9^. Now ${\hat{j}}_z = \sigma _z/2$ ($\sigma _z$ is the Pauli matrix), and the spin’s initial direction is along the *x*-axis, whose azimuthal and polar angles are $\phi =0$ and $\theta =\pi /2$ respectively. According to (21) the final (unnormalised) state of the spin is given by

29 $$\begin{aligned} |\gamma (T_{total}){\rangle }=2^{-1/2} \begin{pmatrix} \int _0^{T_{total}}d\tau A(x\leftarrow G_0|\tau )\exp (-i\omega _L\tau /2)\\ \int _0^{T_{total}}d\tau A(x\leftarrow G_0|\tau )\exp (+i\omega _L\tau /2) \end{pmatrix}\nonumber \\ \approx 2^{-1/2}A(x\leftarrow G_0|\tau ) \begin{pmatrix} 1-i\omega _L{\text {Re}}[{{\overline{\tau }}}]/2 +\omega _L{\text {Im}}[{{\overline{\tau }}}]/2 \\ 1+i\omega _L{\text {Re}}[{{\overline{\tau }}}]/2 -\omega _L{\text {Im}}[{{\overline{\tau }}}]/2) \end{pmatrix}. \end{aligned}$$

As it was discussed in   the third subsection of the Methods, this cannot in general correspond to a rotation around the *z*-axis. On the other hand, in any state, a spin-1/2 must point along some direction on the Bloch sphere. Thus, we expect the state (29) to be rotated not only in the *xy*-, but also in the *xz*-plane. The state of a spin, polarised along a direction making angles $\delta \phi$ and $\pi /2-\delta \theta$ with the *x*- and the *z*-axis, respectively, can be written as

30 $$\begin{aligned} \begin{pmatrix} \exp (-i\delta \phi /2) \cos \left( \frac{\pi }{4}-\frac{\delta \theta }{2}\right) \\ \exp (+i\delta \phi /2) \sin \left( \frac{\pi }{4}-\frac{\delta \theta }{2}\right) \end{pmatrix} \approx 2^{-1/2} \begin{pmatrix} 1-i\delta \phi /2+\delta \theta /2\\ 1+i\delta \phi /2-\delta \theta /2) \end{pmatrix}. \end{aligned}$$

Comparing (29) with (30) we find that the spin has rotated by the (small) angles

31 $$\begin{aligned} \delta \phi = \omega _L{\text {Re}}[{{\overline{\tau }}}], \quad {\text{(in }} {\text{ the }}~ xy~{\text {-plane)}} \quad {\text {and}} \quad \delta \theta = \omega _L{\text {Im}}[{{\overline{\tau }}}] \quad {\text{(in }} {\text{ the }}~ xz~{\text {-plane)}}. \end{aligned}$$

We recall further that a spin travelling with a *classical* particle along a trajectory $x_{class}(t)$ would rotate *only* in the *xy*-plane by an angle $\omega _L\tau _{class}= {\tau _{SW}}[x_{class}(t)]$. Thus, the first of Eq. (31) looks like the classical result, with $\tau _{class}$ replaced by ${\text {Re}}[{{\overline{\tau }}}]$. The second of Eq. (31) has no classical analogue, and should serve as a warning that a straightforward extension of the classical duration to the quantum case may not be possible. (One already knows this from the Uncertainty Principle.)

The appearance of not one, but two rotation angles was first noted by Büttiker in^12^, albeit in a slightly different context. [Ref.^12^ considered transmission of a particle with a known momentum $p_0$ which, in our language, corresponds to replacing $A(x\leftarrow G_0|\tau )$ with $A(p_0\leftarrow G_0|\tau )\equiv \int \exp (-ip_0x)A(x\leftarrow G_0|\tau )dx$ in all formulae, and making $G_0$ nearly monochromatic.] In^12^ Büttiker defined two “times”, $\tau _y \equiv \delta \phi /\omega _L$ and $\tau _z \equiv \delta \theta /\omega _L$, which correspond to our $R[{{\overline{\tau }}}]$ and ${\text {Im}}[{{\overline{\tau }}}]$, respectively. Ramos *et al* measured both the real and the imaginary parts of ${{\overline{\tau }}}$, which can be seen in Fig. 3 of^9^. The authors of^9^ found both parameters positive and concluded that their results were “inconsistent with claims that tunnelling takes ’zero time’”. To abide by this conclusion one needs to take for granted that the “time tunnelling takes” exists as a meaningful concept, but this is not the case.

The confusion can be traced back to Büttiker^12^. When faced with two times parameters instead of one, he opted for a non-negative combination of the two, $\tau _x \equiv \sqrt{\tau _y^2+\tau _z^2}$. This equals the modulus of the “complex time” in Eq. (27), $\tau _x=|\overline{\tau }|$. At least one point made in^12^ requires a comment, if not a correction. In $\tau _x$ Büttiker believed to have found (we read in the Abstract of^12^) “the time interval during which a particle interacts with the barrier if it is finally transmitted.” However, neither ${\text {Re}}[{{\overline{\tau }}}]$ nor ${\text {Im}}[{{\overline{\tau }}}]$, nor any combination of the two can be interpreted as a physical time interval. A weighted sum of quantum mechanical amplitudes, ${{\overline{\tau }}}$, may not give a meaningful answer to the question “how much time does a tunnelling particle spend within the barrier region?” for the same reason the Uncertainty Principle^3^ forbids identifying the particle’s path in Young’s double-slit experiment.
